# Representation of Medical Concepts in Emojis Using Medical Subject Headings to Identify Gaps and Opportunities: Cross-Sectional Analysis

**DOI:** 10.2196/70130

**Published:** 2025-10-14

**Authors:** Shuhan He, Boyu Peng, Suhanee Mitragotri, Ahmad Hassan, Abdel Badih el Ariss, Margarita Monge, Norawit Kijpaisalratana

**Affiliations:** 1 Department of Emergency Medicine, Massachusetts General Hospital, 55 Fruit Street, Boston, MA, 02114-2621, United States; 2 Massachusetts General Hospital, Institute of Health Professions, Boston, MA, United States; 3 Department of Molecular and Cellular Biology, Harvard University, Cambridge, MA, United States; 4 Department of Psychiatry, University of California, Riverside, Riverside, CA, United States

**Keywords:** emoji, health care communication, language, artificial intelligence in health care, digital health tools

## Abstract

**Background:**

Emoji are a universal visual language widely used in digital communication; yet, their representation of medical concepts remains limited. The introduction of medical emojis, such as the anatomical heart and lungs, highlights their potential for health care communication, but significant gaps persist.

**Objective:**

This study aims to systematically analyze the representation of medical concepts in emoji by mapping Medical Subject Headings (MeSH) to Unicode emojis, identifying gaps in medical emoji representation, and proposing areas for future emoji development.

**Methods:**

A cross-sectional study was conducted using the sentence transformer model. Digital resources, including the MeSH thesaurus and Unicode emoji set version 15.0 (Unicode Consortium), were used. Embeddings for 2077 MeSH terms and 3055 emojis were generated, and cosine similarity scores were calculated to evaluate the semantic alignment between MeSH terms and emoji descriptions. A threshold of 0.7 was set to indicate a strong semantic match.

**Results:**

The analysis revealed significant variations in emoji representation across medical categories. “Geographicals” had the highest match rate (33.33%), whereas “Anatomy” showed only 7.94% matches, with 13 of 163 terms exceeding the similarity threshold. Categories such as “Disciplines and Occupations,” “Information Science,” and “Psychiatry and Psychology” had no matches (0%), highlighting notable gaps. The findings underscore substantial disparities in medical emoji representation, particularly for internal organs, mental health, and specialized disciplines. Limited availability of representative emoji may hinder effective health care communication, especially in digital health contexts. This study emphasizes the potential of artificial intelligence to design emojis that address these gaps and improve inclusivity.

**Conclusions:**

Significant gaps in medical emoji representation across various domains were identified. Future efforts should prioritize underrepresented medical categories and leverage artificial intelligence–driven approaches for emoji development to enhance health care communication and accessibility.

## Introduction

### Background

Overall, 92% of the world’s population uses emoji [1], resulting in around 2‐3 trillion emojis being sent in text messages annually [2]. Emojis are built on Unicode, a universal technical standard that supports multiple languages, including other visual languages such as Chinese and Arabic, as well as English symbols, enabling cross-channel communication across devices worldwide, including PCs, Macs, mobile devices, and almost all electronic medical records today.

The Unicode Consortium, a nonprofit organization, accepts proposals for new emojis, allowing the general public to indicate what important concepts should be represented in emojis [3].

The release of Unicode version 13.0 (Unicode Consortium) in March 2020 included anatomical representations of the heart and lungs, which was a substantial development in expanding the range of medically relevant emojis that already existed, such as the stethoscope, syringe, and pill emojis.

These new emojis, due to their integration into the universal digital standard, enhance the representation of the medical field on online technology platforms and are useful for cross-linguistic communication, creating semantic meaning for machine learning analysis, and improving visual analog scales for patient-reported outcomes [4].

However, further progress for a more comprehensive set of medical emojis including the liver, stomach, and kidney has faced significant barriers since 2020 with rejections of these proposals. The reasons for these rejections remain publicly undisclosed, but may be attributed to insufficient evidence of necessity, lack of a unifying standard for medical emojis, and the absence of an objective measure for determining the need for specific health care emojis. These rejections underscore the need to evaluate the scientific evidence for their inclusion, particularly the importance of a comprehensive framework to determine which medical concepts are needed to fill the gaps in the emoji Unicode standard.

### Objectives

The Unified Medical Language System (UMLS) serves as a repository for medical terminology, classification, and coding standards, and it improves the ability of computers to understand the clinical meaning in user inquiries for health information. As a standard for encoding health-related concepts, the UMLS provides a comprehensive framework to better understand potential gaps in Unicode emojis. Thus, we aimed to comprehensively analyze UMLS terms in relation to all Unicode emojis to identify gaps and ensure the proper representation of key health care concepts in the Unicode standard.

## Methods

### Overview

This study used a cross-sectional design to systematically analyze and map the representation of medical concepts using emojis. Our methodology involved integrating digital resources including the Medical Subject Headings (MeSH) thesaurus and the Unicode emoji set version 15.0 (Unicode Consortium).

### Data Preparation

#### MeSH Terms

We use the MeSH thesaurus, a controlled and hierarchically organized vocabulary curated by the National Library of Medicine, for indexing, cataloging, and searching biomedical and health-related information.

MeSH is structured in multiple hierarchical levels, typically up to 12, allowing for very specific indexing and searching of topics. For our analysis, we focused on the top 2 layers, comprising 2077 terms. This selection was made to balance the depth and breadth of the vocabulary, ensuring comprehensive coverage while maintaining manageable complexity.

#### Emoji

The emoji set from version 15.0, comprising 3055 emojis, was used in our analysis [5]. For each emoji, we used its formal Unicode Technical Committee (UTC) name as the textual representation. Examples of these formal UTC names include “grinning face” (for 😀), “red heart” (for ❤️), and “thumbs up” (for 👍). We selected the UTC name rather than the emoji glyph (image) because sentence transformer models require textual input. This approach follows best practices in natural language processing for emoji modeling. Notably, Eisner et al [6] demonstrated that short textual descriptions of emoji can yield high-quality semantic representations, outperforming usage-derived embeddings in many cases [6]. While visual features likely carry additional semantic information, integrating image-based models (eg, CLIP [Contrastive Language-Image Pretraining]) would require a fundamentally different, multimodal pipeline and falls outside the scope of this text-embedding study. Our approach provides a reproducible, text-only baseline for evaluating medical concept alignment.

### Model Selection

For our analysis, we used a high-performing sentence transformer model selected from the Massive Text Embedding Benchmark (MTEB) [7], a comprehensive framework designed to evaluate the performance of text-embedding models across a diverse range of tasks. Specifically, we chose the “mxbai-embed-large-v1” model [8], an open-source solution that consistently ranks among the top 10 models for semantic textual similarity performance. This model was selected not only for its exceptional accuracy but also for its efficiency in terms of computational resources. Its lightweight nature ensures accessibility and practicality, facilitating easy replication of our analysis while maintaining high standards of performance.

### Calculate Embeddings

Sentence embedding involves converting each sentence or textual input into a continuous vector, with the objective of capturing the semantic meaning and contextual nuances in a distributed representation [9-11]. Using the selected model, we generated embeddings for each emoji description and each MeSH term definition. This process yielded a vector for every entity, effectively representing their semantic attributes in numerical form. For example, “Heart: The hollow, muscular organ that maintains the circulation of the blood” will be converted into the following array: [−0.12453227, −0.12998965, 0.00507377, 0.19754894, −0.4001878, 0.2854124, 0.07951714,...−0.12513563, −0.07079408, −0.17973363, 0.20297979, 0.4145004, 0.01179076]

The shape of the array depends on the model used to convert the text; in the case of the “mxbai-embed-large-v1” model, the shape is (512,).

### Calculate Similarity

After transforming tokens into vectors through the embedding process, we use cosine similarity to measure the angle between 2 vectors. This metric determines the similarity between each MeSH term and emoji, producing a score ranging from −1 to 1 for each pair. A score close to −1 indicates that the vectors are diametrically opposed, meaning the words are completely unrelated and oppositely related in the vector space. A score close to 0 suggests the vectors are orthogonal, indicating no linear relationship and that the words are unrelated or independent in the context of the vector space. A score close to 1 means the vectors are identical in direction, signifying that the words are very similar or essentially the same in the vector space [12,13].

The cosine similarity between 2 vectors A and B is calculated using the formula:


$$\text{similarity}(\underline{A},\underline{B})=(A\cdot B)/(\|A\|\times \|B\|)$$

where A · B represents the dot product of vectors A and B, and ||A|| and ||B|| represent the magnitudes (Euclidean norms) of vectors A and B, respectively. This can also be expressed as:


$$\text{similarity}(A,B)=\sum (A_{i}\times B_{i})/(\sqrt{\sum (A_{i}^{2})}\times \sqrt{\sum (B_{i}^{2})})$$

where Ai and Bi are the *i*th components of vectors A and B, and the summations are from *i*=1 to n, with n being the dimension of the vectors. We adopted a cosine similarity threshold of 0.7 as the criterion for a match, indicating a strong similarity between an emoji and a MeSH term. All analyses were performed using Python version 3.10.12 (Python Software Foundation [PSF]).

### Statistical Analysis

To assess differences in cosine similarity scores across various MeSH categories, we used the Kruskal-Wallis H test, a nonparametric method suitable for nonnormally distributed data. This test identifies statistically significant differences in group medians. When such differences were found, we performed the Dunn test for pairwise comparisons, applying the Bonferroni correction to adjust for multiple testing. All statistical analyses were conducted using Stata (version 18.0; StataCorp LLC).

### Ethical Considerations

This study did not involve human participants, patient data, or identifiable personal information. Therefore, institutional review board approval and informed consent were not required. All data used were publicly available and nonidentifiable, and the research adhered to relevant institutional and national guidelines regarding privacy and ethical conduct. No compensation was provided, as no participants were recruited or involved.

## Results

**Figure 1.**
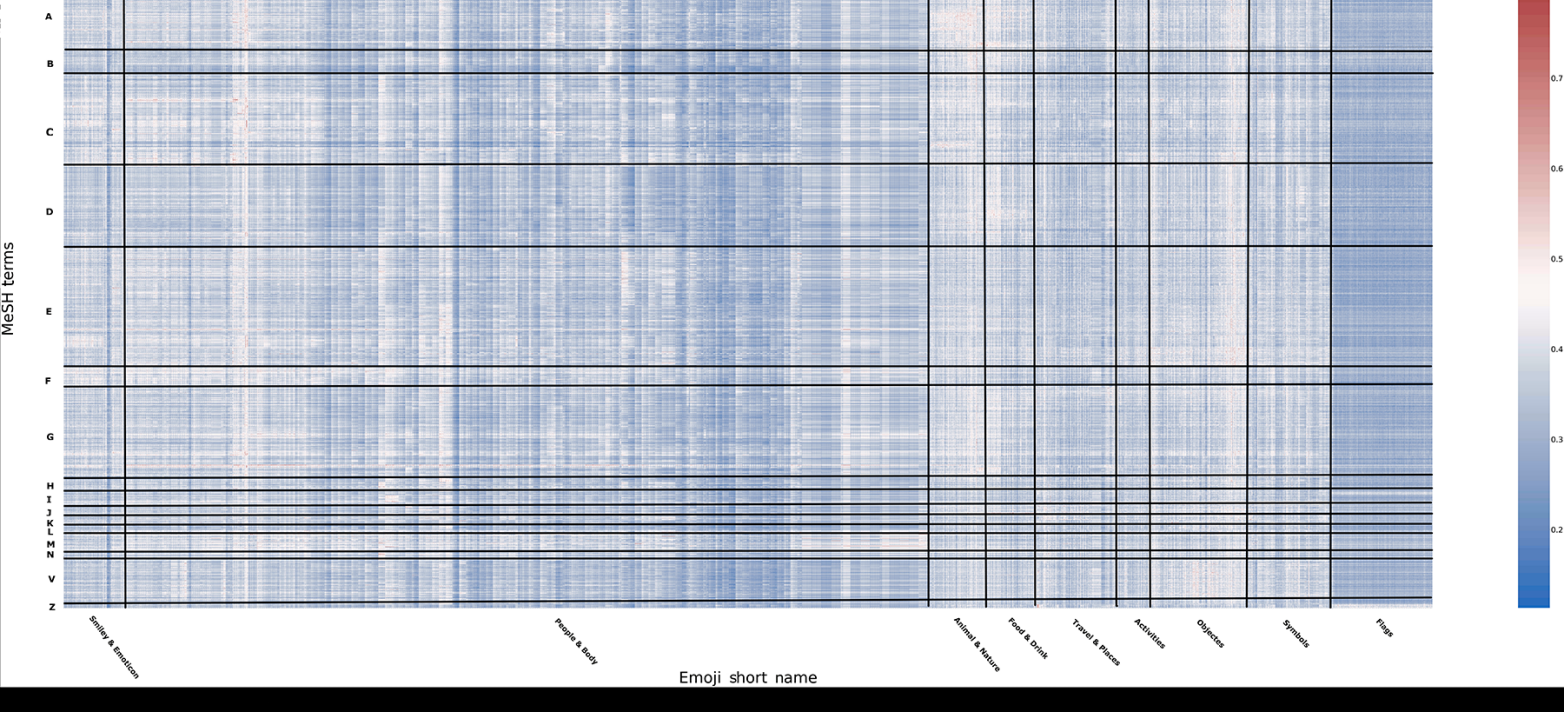
Heatmap of cosine similarity scores between Medical Subject Headings terms and emojis. Medical Subject Headings terms are categorized as follows: A=Anatomy; B=Organisms; C=Diseases; D=Chemicals and Drugs; E=Analytical, Diagnostic, and Therapeutic Techniques, and Equipment; F=Psychiatry and Psychology; G=Phenomena and Processes; H=Disciplines and Occupations; I=Anthropology, Education, Sociology, and Social Phenomena; J=Technology, Industry, and Agriculture; K=Humanities; L=Information Science; M=Named Groups; N=Health Care; V=Publication Characteristics; Z=Geographicals. MeSH: Medical Subject Headings.

Our analysis generated embeddings for 2077 MeSH terms and 3055 emojis, resulting in over 6 million similarity comparisons. Figure 1 illustrates the heatmap of cosine similarity scores between MeSH terms and emoji. Each pixel represents a similarity score between a specific MeSH term and an emoji. The color scale ranges from blue (low similarity) to red (high similarity), and grid lines segment the MeSH terms and emojis into categories to enhance pattern visibility. The predominance of blue shades indicates that the majority of MeSH terms and emoji pairs have low cosine similarity scores, suggesting that most emojis do not closely match specific medical terms in the MeSH dataset. The sparsity of red or high similarity scores highlights that only a small subset of emojis aligns well with medical terminology.

The analysis of cosine similarity scores across MeSH categories mapped to Unicode emojis highlighted significant variability (Table 1).

The median cosine similarity score in the “Geographicals (Z)” category was 0.70 (IQR 0.64‐0.74), indicating a strong alignment between MeSH terms and emojis within this category. This category also showed statistically significantly higher scores compared to all other categories, with *P* values <.001 in each comparison. This was followed by “Diseases (C)” with a median score of 0.68 (IQR 0.55‐0.62) and “Technology, Industry, and Agriculture (J)” with a median of 0.63 (IQR 0.58‐0.67). In contrast, “Chemicals and Drugs (D)” recorded the lowest median score at 0.56 (IQR 0.54‐0.59), suggesting a relatively weaker representation of these concepts in emoji form. Categories such as “Anthropology, Education, Sociology, and Social Phenomena (I)” and “Psychiatry and Psychology (F)” also demonstrated lower median scores.

Table 2 presents the top 10 MeSH terms and emoji pairs with the highest similarity scores, ranging from 0.7747 to 0.8198. The highest similarity score of 0.8198 was observed between “Seedlings” and the seedling emoji (🌱), followed by “Grandparents” matched with the family emoji (👨‍👩‍👧‍👦) at 0.8128, and “Nose” with the nose emoji (👃) at 0.8008. The matched pairs encompass various concepts, including anatomical parts (“Nose”), geographical entities (“Europe”), social concepts (“Grandparents”), facilities (“Schools,” “Bathroom Equipment,” “Toilet Facilities”), and scientific tools (“Microscopy,” “Syringes”). Most pairs show direct correspondence between the MeSH term and the emoji, such as “Syringes” with the syringe emoji (💉) and “Schools” with the school emoji (🚻). Two MeSH terms, “Bathroom Equipment” and “Toilet Facilities,” were both matched with the same restroom emoji (🏫), with similarity scores of 0.7769 and 0.7747, respectively. The geographical concept “Europe” was matched with the “globe showing Europe-Africa” emoji (🌍) with a similarity score of 0.7907. All MeSH terms and emoji pairs exceeding the similarity threshold are presented in Table S1 and Table S2 in Multimedia Appendix 1.

In the analysis of human anatomy-related MeSH terms and emoji pairs (Table 3), 13 pairs exceeded the similarity threshold, with scores ranging from 0.7014 to 0.8008. The “Nose” and nose emoji (👃) pair maintained its high ranking from the overall top 10, with a score of 0.8008. The mouth emoji (👄) corresponded to multiple anatomical terms: “Mouth” (0.7532), “Stomatognathic System” (0.7400), and “Jaw” (0.7175). Similarly, the bone emoji (🦴) is paired with both “Musculoskeletal System” (0.7353) and “Skeleton” (0.7202). Internal organs were also represented, with “Lung” matching the lungs emoji (🫁) at 0.7555 and “Heart” matching the heart emoji (🫀) at 0.7329.

**Table 1.** Median cosine similarity scores for each Medical Subject Headings category and intercategory comparisons.

		Median (IQR)	A^a^	B^b^	C^c^	D^d^	E^e^	F^f^	G^g^	H^h^	I^i^	J^j^	K^k^	L^l^	M^m^	N^n^	V^o^	Z^p^
A		0.60 (0.57-0.64)																
	*r*		1	1.98	12.12	24.78	4.65	11.02	7.76	3.75	10.34	−4.35	6.49	4.90	1.30	12.31	4.88	−13
	*P* value		—^q^	>.99	<.001	<.001	<.001	<.001	<.001	.01	<.001	<.001	<.001	<.001	>.99	<.001	<.001	<.001
B		0.60 (0.57-0.62)																
	*r*		1.98	1	8	18.58	1.82	8.24	4.78	2.21	8.47	−5.46	5.43	3.64	0.06	9.30	3.60	−13.7
	*P* value		>.99	—	<.001	<.001	>.99	<.001	<.001	>.99	<.001	<.001	<.001	.02	>.99	<.001	.02	<.001
C		0.68 (0.55-0.62)																
	*r*		12.12	8	1	17.91	−9.01	2.55	−3.40	−2.84	4.24	−11.20	2.37	−0.19	−4.46	3.73	−0.30	−19.90
	*P* value		<.001	<.001	—	<.001	<.001	.64	.04	.27	.01	<.001	>.99	>.99	<.001	.01	>.99	<.001
D		0.56 (0.54-0.59)																
	*r*		24.78	18.58	17.91	1	−2.51	−7.83	−1.70	−9.76	−2.65	−17.8	−1.94	−5.52	−10.30	−7.17	−5.72	−26.21
	*P* value		<.001	<.001	<.001	—	<.001	<.001	<.001	<.001	.48	<.001	>.99	<.001	<.001	<.001	<.001	<.001
E		0.59 (0.56-0.63)																
	*r*		4.65	1.82	−9.01	−2.51	1	8.26	4.09	1.21	8.15	−7.19	4.89	2.92	−0.99	9.64	2.86	−16
	*P* value		<.001	>.99	<.001	<.001	—	<.001	.01	>.99	<.001	<.001	<.001	.21	>.99	<.001	.25	<.001
F		0.57 (0.54-0.61)																
	*r*		11.02	8.24	2.55	−7.83	8.26	1	−4.66	−4.01	2.23	−11.40	1.23	−1.40	−5.39	0.80	−1.52	−19.40
	*P* value		<.001	<.001	.64	<.001	<.001	—	<.001	.01	>.99	<.001	>.99	>.99	<.001	>.99	>.99	<.001
G		0.58 (0.55-0.62)																
	*r*		7.76	4.78	−3.40	−1.70	4.09	−4.66	1	−1.01	5.71	–9.06	3.41	1.14	−2.85	5.78	1.06	−17.60
	*P* value		<.001	<.001	.04	<.001	.01	<.001	—	>.99	<.001	<.001	.04	>.99	.26	<.001	>.99	<.001
H		0.58 (0.56-0.62)																
	*r*		3.75	2.21	−2.84	−9.76	1.21	−4.01	−1.01	1	5.22	−6.41	3.60	1.63	−1.62	4.68	1.57	−13.50
	*P* value		.01	>.99	.27	<.001	>.99	.01	>.99	—	<.001	<.001	.02	>.99	>.99	<.001	>.99	<.001
I		0.56 (0.54-0.60)																
	*r*		10.34	8.47	4.24	−2.65	8.15	2.23	5.71	5.22	1	−11.50	−0.25	−2.83	−6.37	−1.67	−2.95	−18.40
	*P* value		<.001	<.001	.01	.48	<.001	>.99	<.001	<.001	—	<.001	>.99	.28	<.001	>.99	.19	<.001
J		0.63 (0.58-0.67)																
	*r*		−4.35	−5.46	−11.20	−17.8	−7.19	−11.40	-9.06	−6.41	−11.50	1	8.33	7.12	4.27	12.18	7.12	−7.09
	*P* value		<.001	<.001	<.001	<.001	<.001	<.001	<.001	<.001	<.001	—	<.001	<.001	.001	<.001	<.001	<.001
K		0.57 (0.54-0.60)																
	*r*		6.49	5.43	2.37	−1.94	4.89	1.23	3.41	3.60	−0.25	8.33	1	−2	−4.66	−0.84	−2.08	−13.70
	*P* value		<.001	<.001	>.99	>.99	<.001	>.99	.04	.02	>.99	<.001	—	>.99	<.001	>.99	>.99	<.001
L		0.58 (0.55-0.62)																
	*r*		4.90	3.64	−0.19	−5.52	2.92	−1.40	1.14	1.63	−2.83	7.12	−2.00	1	−2.94	1.90	−0.07	−13.30
	*P* value		<.001	.02	>.99	<.001	.21	>.99	>.99	>.99	.28	<.001	>.99	—	.20	>.99	>.99	<.001
M		0.60 (0.55-0.65)																
	*r*		1.30	0.06	−4.46	−10.30	−0.99	−5.39	−2.85	−1.62	−6.37	4.27	−4.66	−2.94	1	5.98	2.89	–10.8
	*P* value		>.99	>.99	<.001	<.001	>.99	<.001	.26	>.99	<.001	.001	<.001	.20	—	<.001	.23	<.001
N		0.57 (.54-.61)																
	*r*		12.31	9.30	3.73	−7.17	9.64	0.80	5.78	4.68	−1.67	12.18	−0.84	1.9	5.98	1	−2.02	–20.2
	*P* value		<.001	<.001	.01	<.001	<.001	>.99	<.001	<.001	>.99	<.001	>.99	>.99	<.001	—	>.99	<.001
V		0.58 (.55- .61)																
	*r*		4.88	3.60	−0.30	−5.72	2.86	−1.52	1.06	1.57	−2.95	7.12	−2.08	−0.07	2.89	−2.02	1	–13.3
	*P* value		<.001	.02	>.99	<.001	.25	>.99	>.99	>.99	.19	<.001	>.99	>.99	.23	>.99	—	<.001
Z		0.70 (.64-.74)																
	*r*		−13.00	−13.7	−19.90	−26.21	−16.00	−19.40	−17.60	−13.50	−18.40	−7.09	−13.70	−13.30	–10.80	–20.20	–13.30	1
	*P* value		<.001	<.001	<.001	<.001	<.001	<.001	<.001	<.001	<.001	<.001	<.001	<.001	<.001	<.001	<.001	—

a A: Anatomy.

b B: Organisms.

c C: Diseases.

d D: Chemicals and Drugs.

e E: Analytical, Diagnostic and Therapeutic Techniques, and Equipment.

f F: Psychiatry and Psychology.

g G: Phenomena and Processes.

h H: Disciplines and Processes.

i I: Anthropology, Education, Sociology, and Social Phenomena.

j J: Technology, Industry, and Agriculture.

k K: Humanities.

l L: Informatics Science.

m M: Named Groups.

n N: Health Care.

o V: Publication Characteristics.

p Z: Geographicals.

q Not applicable.

**Table 2.** Top 10 Medical Subject Headings terms and emoji pairs with the highest similarity.

MeSH^a^ heading	Unicode CLDR^b^ short name	Emoji	Cosine similarity score
Seedlings	seedling	🌱	0.8198
Grandparents	family: man, woman, girl, boy	👨‍👩‍👧‍👦	0.8128
Nose	nose	👃	0.8008
Europe	globe showing Europe-Africa	🌍	0.7907
Pedestrians	person walking	🚶	0.7833
Schools	school	🏫	0.7827
Syringes	syringe	💉	0.7801
Microscopy	microscope	🔬	0.7776
Bathroom equipment	restroom	🚻	0.7769
Toilet facilities	restroom	🚻	0.7747

a MeSH: Medical Subject Headings.

b CLDR: Common Locale Data Repository

**Table 3.** Human anatomy Medical Subject Headings term and emoji pairs exceeding the similarity threshold.

MeSH^a^ heading	Emoji	Cosine similarity score
Nose	👃	0.8008
Lung	🫁	0.7555
Mouth	👄	0.7532
Eye	👁️	0.7408
Stomatognathic system	👄	0.7400
Musculoskeletal system	🦴	0.7353
Heart	🫀	0.7329
Ear	👂	0.7260
Cheek	🫤	0.7233
Skeleton	🦴	0.7202
Jaw	👄	0.7175
Central nervous system	🧠	0.7172
Acinar cells	🫁	0.7014

a MeSH: Medical Subject Headings.

**Figure 2.**
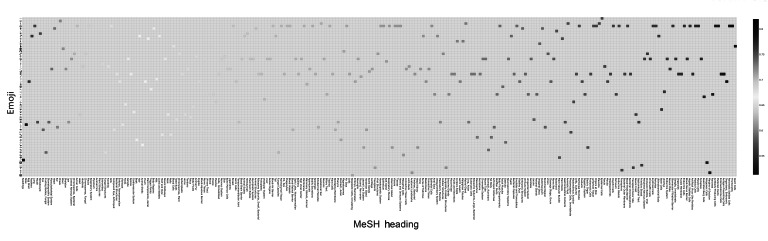
Heatmap of cosine similarity scores between anatomical Medical Subject Headings terms and emojis. MeSH: Medical Subject Headings.

The heatmap visualizes cosine similarity scores between anatomical MeSH terms and emojis, and colors the cell with the highest score (Figure 2). The x-axis lists anatomical MeSH sorted from highest to lowest match pairs, while the y-axis represents different emojis. A color scale from dark blue (low similarity) to dark red (high similarity) is used, with white and light shades representing intermediate values. The majority of the heatmap exhibits light blue colors, indicating generally low similarity scores between most anatomical terms and emoji. For full figure access, refer to [14].

Table S1 in Multimedia Appendix 1 reveals significant gaps in emoji representation for human anatomical terms, with unmatched pairs highlighted in red. Out of 163 terms examined, 150 (92.02%) did not meet the established similarity threshold. Several vital organs show notably low similarity scores. The pancreas at 0.5293 and the liver at 0.6285. The urogenital system scored 0.5877, with genitalia and urinary tract at 0.5875 and 0.6441, respectively. The endocrine system exhibited one of the lowest scores at 0.5539. The cardiovascular system scored 0.6682, with the heart as an exception at 0.7329. The nervous system scored 0.6395, showing a notable discrepancy between its central (0.7172) and peripheral (0.5598) components.

Table 4 shows the percentage of matching MeSH top-level categories with corresponding emojis. “Geographicals” has the highest match rate at 33.33%, followed by “Named Groups” (11.94%) and “Technology, Industry, and Agriculture” (11.36%). Most categories have less than 10% matches, with “Anatomy” at 7.94%. “Organisms” (1.35%) and “Chemicals and Drugs” (0.84%) show very few matches. Three categories—“Disciplines and Occupations,” “Information Science,” and “Psychiatry and Psychology”—have no matches (0%). The matching percentages range from 0% to 33.33%, revealing significant variation in emoji representation across medical and scientific domains.

**Table 4.** Percentage of matching Medical Subject Headings top-level categories with corresponding emojis.

MeSH^a^ top-level categories	Percentage of matching
Geographicals	33.33
Named Groups	11.94
Technology, Industry, and Agriculture	11.36
Anatomy	7.94
Humanities	7.69
Analytical, Diagnostic and Therapeutic Techniques, and Equipment	5.31
Health Care	5
Diseases	4.66
Phenomena and Processes	4.08
Publication Characteristics	3.25
Anthropology, Education, Sociology, and Social Phenomena	1.82
Organisms	1.35
Chemicals and Drugs	0.84
Disciplines and Occupations	0
Information Science	0
Psychiatry and Psychology	0

a MeSH: Medical Subject Headings.

## Discussion

### Principal Findings

Unicode regularly introduces new figures relevant to medicine, reflecting the growing importance of visual communication in health care. Recent additions such as the anatomical heart (🫀) and lungs (🫁) emoji [15] demonstrate this commitment. Our analysis revealed high similarity scores between these anatomical emojis and their corresponding MeSH terms (heart: 0.7329, lung: 0.7555), indicating their effectiveness in representing these concepts. However, these additions are just the beginning of what could be a more comprehensive set of medical emojis. However, our study was limited to text-based descriptions of emojis and did not incorporate the emoji glyphs themselves. Emoji glyphs often contain rich visual features—such as stylization, implied movement, or symbolic elements—that are not fully captured in their official Unicode names. Future work could incorporate visual-semantic models such as CLIP, which align text and images in a shared embedding space and have been shown to capture multi-modal meaning with high fidelity (Radford et al [16]). Such an approach could improve alignment between medical terms and emoji representations, particularly in anatomy and other visually grounded domains.

Our research identified significant variations in emoji-MeSH term matching across categories, highlighting uneven visual representation in medical communication. The “Geographicals” category showed the highest match rate at 33.33%, likely due to universally recognizable symbols for locations and features. This finding highlights a broader pattern in emoji design and use. The high alignment observed in the “Geographicals” category is likely due to the iconic and visually intuitive nature of geographical emojis, such as globe symbols (🌍), maps (🗺️), and flags (🏁), which are easily associated with MeSH terms such as “Europe” or “Mountains.” These emojis rely on universally understood visual semantics, enabling higher alignment with general-language terms. In contrast, many medical concepts—especially internal anatomical structures or abstract scientific terms—lack such iconic visual representations in the Unicode emoji set. This reflects a design bias in the current emoji lexicon toward common, general-purpose concepts, rather than specialized domains such as healthcare or biomedical science. As a result, while categories such as geography are well represented, critical areas of medicine remain visually underrepresented. This discrepancy supports the central hypothesis of our study: that emoji coverage is uneven and that systematic gaps exist in the representation of medical and health-related terminology. Conversely, categories such as “Disciplines and Occupations,” “Information Science,” and “Psychiatry and Psychology” had no matches. This absence underscores a critical gap in representing complex and abstract medical and scientific concepts. For instance, “Disciplines and Occupations” includes various medical specialties lacking specific visual symbols, while “Information Science” involves nonvisual concepts like data science. The lack of matches in “Psychiatry and Psychology” is particularly significant, given the increasing importance of mental health discussions. This gap may hinder effective communication and destigmatization efforts in these fields.

These findings are further substantiated by our detailed analysis of anatomical terms, as shown in Table S1 in Multimedia Appendix 1. Specific anatomical structures or medical equipment that currently lack emoji representations should be prioritized. For example, organs such as the kidney, liver, and pancreas are fundamental to many medical discussions yet lack direct emoji representations. The urogenital system and endocrine system also demonstrate poor representation. Interestingly, while some external features, such as the nose and mouth, have high similarity scores, internal organs and systems generally lack appropriate emoji matches. The need for these emoji is supported by previous calls from professional medical societies, such as the American Society of Nephrology’s advocacy for a kidney emoji [17]. Our study reinforces this need, as evidenced by the low matching percentage in the “Anatomy” category (7.94%) and the absence of these specific organs in our high-similarity matches. This discrepancy highlights the need for a more balanced approach in developing medical emojis, ensuring that both visible and internal anatomical structures are adequately represented to enhance medical communication and education.

Beyond their role in medical terminology representation, emojis offer unique usability advantages in health care communication. Images and symbols can be processed more quickly than text, making emojis potentially valuable for rapid information transfer in medical informatics. This is particularly relevant in environments such as clinical documentation, emergency communication, and patient engagement tools, where quick comprehension is essential. Automation and usability improvements—such as integrating emoji into electronic health records or artificial intelligence (AI)–driven clinical decision support systems—could further enhance their role in medical communication. However, for emojis to be effectively used in health care settings, their medical relevance and standardization must be improved.

This need for more comprehensive medical emoji representation aligns with the increasing interest in using emoticons and emojis in health care communication over the past couple of years. Emojis have been explored to be useful in public health communication, such as conveying important hand hygiene and infection control protocols to the public [18,19]. Emojis have also been used to develop novel scales for patients to use to communicate their mood, pain, or satisfaction with clinical care [20]. Research has shown that emojis also show promise in provider-provider communication in secure clinical texting systems, conveying new and salient information [21]. The universality of the use of emoji is indicative of their utility and value in the medical field.

### Significance

While traditional emoji creation processes continue, new approaches could further enhance the representation of medical concepts. The rapid advancement of generative AI in health care, capable of creating enhanced medical images and facilitating drug design discovery [22,23], presents exciting possibilities for emoji development. Leveraging these AI capabilities, we could address the need for more diverse and specific medical emojis through dynamic AI generation and sticker-based systems. AI-generated emojis could offer a wider range of customizable medical symbols, adapting to specific needs in real time, while complementary sticker-based approaches could provide greater flexibility in representing complex medical concepts. These innovative methods could significantly enhance the visual communication of medical information, bridging the gaps we identified in the current emoji set and providing more tailored options for health-related discussions.

Future research could build upon our findings through several complementary enhancements. First, domain-specific language models such as BioBERT or SciBERT could be used to generate embeddings that more accurately capture biomedical semantics, potentially reducing false positives and improving MeSH–emoji alignment for clinically nuanced terms. Second, multimodal approaches, such as CLIP [16], could enable direct comparison between MeSH terms and the visual glyphs of emoji. This would allow researchers to evaluate how visual meaning, beyond textual labels, might influence semantic alignment—particularly for categories such as anatomy where appearance plays a critical role in interpretation. Together, these strategies could substantially enhance the resolution and accuracy of concept-emoji mapping in future work. By implementing these approaches, we could potentially address the disparity observed in emoji representation across different medical and scientific domains, moving toward a more comprehensive and nuanced visual language for health care communication. One promising direction involves integrating visual-semantic models such as CLIP to directly compare emoji glyphs to medical concepts. This could allow more accurate alignment for visual categories such as anatomy, where symbolic form and visual context are essential for meaning.

### Limitations

This study has several limitations that should be considered when interpreting the results. First, our analysis was based on cosine similarity scores between MeSH terms and emoji descriptions, which may not capture all nuances of semantic relationships. The effectiveness of emoji in real-world medical communication may differ from these computational assessments. Second, we focused on the top 2 layers of MeSH terms, which, while providing a broad overview, may have excluded more specific medical concepts that could benefit from emoji representation. Third, MeSH terminology is highly domain-specific, while emoji descriptions tend to be more general, leading to potential mismatches in similarity scores. This discrepancy may affect the accuracy of identifying true gaps in medical emoji representation. Similarly, the high match rate in the “Geographicals” category suggests that universally recognizable symbols may skew similarity scores, making it difficult to distinguish meaningful medical associations.

In addition, our study used a specific sentence transformer model for generating embeddings, and results might vary with different models or embedding techniques. Finally, cultural and regional differences in emoji interpretation were not accounted for in this study, which could impact the global applicability of our findings.

### Conclusion

Our study reveals significant disparities in emoji representation across medical domains, offering evidence-based insights for future development. By prioritizing underrepresented areas and embracing innovative approaches, we can create a more comprehensive set of medical emojis. These advancements have the potential to enhance health care communication, improve patient-provider interactions, and ultimately contribute to better health outcomes.

## Supplementary material

10.2196/70130 Multimedia Appendix 1 Cosine similarity scores between human anatomical MeSH (Medical Subject Headings) terms and corresponding emojis and MeSH Term–emoji pairs exceeding the similarity threshold.

## Abbreviations

AI: artificial intelligence
CLIP: Contrastive Language-Image Pretraining
MeSH: Medical Subject Headings
MTEB: Massive Text Embedding Benchmark
UMLS: Unified Medical Language System
UTC: Unicode Technical Committee
