# Supplementary material for: Representation of Medical Concepts in Emojis Using Medical Subject Headings to Identify Gaps and Opportunities: Cross-Sectional Analysis
Source: JMIR Form Res. 2025 Oct 14;9:e70130. doi: 10.2196/70130 (PMC12520250; doi:10.2196/70130)
Supplement: Multimedia Appendix 1 [file formative-v9-e70130-s001.docx]

**Supplementary Tables S1 and S2**

**Table S1.** Cosine Similarity Scores Between Human Anatomical MeSH Terms and Corresponding Emojis.

| **MeSH Heading** | **MeSH Tree** | **Cosine Similarity Score** |
| --- | --- | --- |
| **Body Regions** | **A01** | **0.6625** |
| Anatomic Landmarks | A01.111 | 0.6094 |
| Breast | A01.236 | 0.634 |
| Extremities | A01.378 | 0.6844 |
| Head | A01.456 | 0.6961 |
| Neck | A01.598 | 0.659 |
| Organs at Risk | A01.635 | 0.5709 |
| Perineum | A01.719 | 0.567 |
| Torso | A01.923 | 0.609 |
| Transplant Donor Site | A01.935 | 0.5648 |
| Transplants | A01.941 | 0.6147 |
| Trigger Points | A01.947 | 0.6648 |
| Viscera | A01.960 | 0.6118 |
| **Musculoskeletal System** | **A02** | **0.7353** |
| Aponeurosis | A02.083 | 0.6231 |
| Cartilage | A02.165 | 0.6147 |
| Fascia | A02.340 | 0.5686 |
| Ligaments | A02.513 | 0.6355 |
| Muscles | A02.633 | 0.6397 |
| Myotendinous Junction | A02.734 | 0.5743 |
| Skeleton | A02.835 | 0.7202 |
| Tendons | A02.880 | 0.6505 |
| **Digestive System** | **A03** | **0.5731** |
| Biliary Tract | A03.159 | 0.6225 |
| Gastrointestinal Tract | A03.556 | 0.5632 |
| Liver | A03.620 | 0.6285 |
| Pancreas | A03.734 | 0.5293 |
| **Respiratory System** | **A04** | **0.6908** |
| Larynx | A04.329 | 0.587 |
| Lung | A04.411 | 0.7555 |
| Nose | A04.531 | 0.8008 |
| Pharynx | A04.623 | 0.6216 |
| Pleura | A04.716 | 0.6911 |
| Respiratory Mucosa | A04.760 | 0.6126 |
| Trachea | A04.889 | 0.6224 |
| **Urogenital System** | **A05** | **0.5877** |
| Genitalia | A05.360 | 0.5875 |
| Urinary Tract | A05.810 | 0.6441 |
| **Endocrine System** | **A06** | **0.5539** |
| Chromaffin System | A06.224 | 0.5943 |
| Endocrine Glands | A06.300 | 0.567 |
| Enteroendocrine Cells | A06.390 | 0.5273 |
| Neurosecretory Systems | A06.688 | 0.5907 |
| **Cardiovascular System** | **A07** | **0.6682** |
| Blood Vessels | A07.015 | 0.6253 |
| Blood-Air Barrier | A07.020 | 0.6278 |
| Blood-Aqueous Barrier | A07.030 | 0.6465 |
| Blood-Brain Barrier | A07.035 | 0.6329 |
| Blood-Nerve Barrier | A07.037 | 0.5466 |
| Blood-Retinal Barrier | A07.040 | 0.6252 |
| Blood-Testis Barrier | A07.045 | 0.5656 |
| Glomerular Filtration Barrier | A07.500 | 0.5516 |
| Glymphatic System | A07.521 | 0.6318 |
| Heart | A07.541 | 0.7329 |
| **Nervous System** | **A08** | **0.6395** |
| Central Nervous System | A08.186 | 0.7172 |
| Ganglia | A08.340 | 0.6711 |
| Nerve Net | A08.511 | 0.5796 |
| Neural Pathways | A08.612 | 0.6598 |
| Neuroglia | A08.637 | 0.6166 |
| Neurons | A08.675 | 0.6226 |
| Peripheral Nervous System | A08.800 | 0.5598 |
| Synapses | A08.850 | 0.5404 |
| **Sense Organs** | **A09** | **0.6284** |
| Ear | A09.246 | 0.726 |
| Eye | A09.371 | 0.7408 |
| Taste Buds | A09.846 | 0.6536 |
| Vestibular System | A09.923 | 0.6286 |
| **Tissues** | **A10** | **0.6116** |
| Body Fluid Compartments | A10.082 | 0.5912 |
| Connective Tissue | A10.165 | 0.6067 |
| Epithelium | A10.272 | 0.613 |
| Exocrine Glands | A10.336 | 0.6016 |
| Lymphoid Tissue | A10.549 | 0.572 |
| Membranes | A10.615 | 0.6239 |
| Nerve Tissue | A10.755 | 0.6445 |
| Organoids | A10.802 | 0.5514 |
| Parenchymal Tissue | A10.806 | 0.6261 |
| Peritoneal Stomata | A10.810 | 0.5735 |
| Margins of Excision | A10.830 | 0.5301 |
| Surgically-Created Structures | A10.850 | 0.6035 |
| **Cells** | **A11** | **0.6183** |
| Acinar Cells | A11.031 | 0.7014 |
| Allogeneic Cells | A11.047 | 0.5323 |
| Antibody-Producing Cells | A11.063 | 0.5285 |
| Antigen-Presenting Cells | A11.066 | 0.5195 |
| Blood Cells | A11.118 | 0.6563 |
| Bone Marrow Cells | A11.148 | 0.6358 |
| Cells, Cultured | A11.251 | 0.5739 |
| Cellular Structures | A11.284 | 0.604 |
| Chromaffin Cells | A11.299 | 0.5592 |
| Connective Tissue Cells | A11.329 | 0.6102 |
| Endocrine Cells | A11.382 | 0.5486 |
| Epidermal Cells | A11.409 | 0.5705 |
| Epithelial Cells | A11.436 | 0.6066 |
| Erythroid Cells | A11.443 | 0.5584 |
| Eukaryotic Cells | A11.450 | 0.5967 |
| Germ Cells | A11.497 | 0.6332 |
| Giant Cells | A11.500 | 0.5131 |
| Hepatic Stellate Cells | A11.561 | 0.5457 |
| Muscle Cells | A11.620 | 0.542 |
| Myeloid Cells | A11.627 | 0.543 |
| Neoplastic Cells, Circulating | A11.642 | 0.6175 |
| Oxyphil Cells | A11.690 | 0.5671 |
| Pancreatic Stellate Cells | A11.700 | 0.5165 |
| Pericytes | A11.710 | 0.5581 |
| Phagocytes | A11.733 | 0.6254 |
| Reed-Sternberg Cells | A11.828 | 0.535 |
| Stem Cells | A11.872 | 0.5547 |
| Thymocytes | A11.900 | 0.5142 |
| **Fluids and Secretions** | **A12** | **0.6008** |
| Amniotic Fluid | A12.098 | 0.5756 |
| Bodily Secretions | A12.200 | 0.6231 |
| Body Fluids | A12.207 | 0.6493 |
| Exudates and Transudates | A12.383 | 0.5894 |
| Feces | A12.459 | 0.6717 |
| Gastrointestinal Contents | A12.519 | 0.5764 |
| Hyalin | A12.580 | 0.5906 |
| Milk | A12.790 | 0.6621 |
| Secretome | A12.895 | 0.5958 |
| **Stomatognathic System** | **A14** | **0.74** |
| Cheek | A14.194 | 0.7232 |
| Facial Muscles | A14.363 | 0.5911 |
| Jaw | A14.521 | 0.7175 |
| Masticatory Muscles | A14.530 | 0.6043 |
| Mouth | A14.549 | 0.7532 |
| Temporomandibular Joint | A14.907 | 0.6332 |
| **Hemic and Immune Systems** | **A15** | **0.6112** |
| Blood | A15.145 | 0.6428 |
| Hematopoietic System | A15.378 | 0.6137 |
| Immune System | A15.382 | 0.588 |
| **Embryonic Structures** | **A16** | **0.5885** |
| Blastomeres | A16.094 | 0.6072 |
| Branchial Region | A16.142 | 0.5838 |
| Cleavage Stage, Ovum | A16.166 | 0.5833 |
| Ectodermal Placodes | A16.216 | 0.6229 |
| Embryo, Mammalian | A16.254 | 0.6168 |
| Fetus | A16.378 | 0.6504 |
| Gastrula | A16.441 | 0.5908 |
| Germ Layers | A16.504 | 0.5996 |
| Gestational Sac | A16.535 | 0.6227 |
| Gubernaculum | A16.551 | 0.5847 |
| Limb Buds | A16.567 | 0.6097 |
| Mesonephros | A16.599 | 0.5961 |
| Morula | A16.615 | 0.5683 |
| Mullerian Ducts | A16.623 | 0.528 |
| Neural Crest | A16.627 | 0.5634 |
| Neural Plate | A16.629 | 0.5526 |
| Neural Tube | A16.630 | 0.5939 |
| Notochord | A16.660 | 0.5773 |
| Ovum | A16.690 | 0.6664 |
| Placenta | A16.710 | 0.5706 |
| Primitive Streak | A16.830 | 0.5613 |
| Pronephros | A16.835 | 0.5667 |
| Urachus | A16.890 | 0.5546 |
| Vitelline Duct | A16.920 | 0.5889 |
| Wolffian Ducts | A16.935 | 0.5914 |
| Zygote | A16.950 | 0.648 |
| **Integumentary System** | **A17** | **0.6048** |
| Hair | A17.360 | 0.667 |
| Nails | A17.600 | 0.6564 |
| Skin | A17.815 | 0.6388 |

**Table S2.** MeSH Term and Emoji Pairs Exceeding the Similarity Threshold.

| **MeSH Heading** | **CLDR Short Name** | **Emoji** | **Cosine Similarity Score** |
| --- | --- | --- | --- |
| Seedlings | seedling | 🌱 | 0.8198 |
| Grandparents | family: man, woman, girl, boy | 👨‍👩‍👧‍👦 | 0.8128 |
| Nose | nose | 👃 | 0.8008 |
| Europe | globe showing Europe-Africa | 🌍 | 0.7907 |
| Pedestrians | person walking | 🚶 | 0.7833 |
| Schools | school | 🏫 | 0.7827 |
| Syringes | syringe | 💉 | 0.7801 |
| Microscopy | microscope | 🔬 | 0.7776 |
| Bathroom Equipment | restroom | 🚻 | 0.7769 |
| Toilet Facilities | restroom | 🚻 | 0.7747 |
| Africa | globe showing Europe-Africa | 🌍 | 0.7717 |
| Music | musical note | 🎵 | 0.7700 |
| Protective Devices | safety vest | 🦺 | 0.7662 |
| Nose Diseases | nose | 👃 | 0.7655 |
| Egg Shell | egg | 🥚 | 0.7651 |
| Feathers | feather | 🪶 | 0.7632 |
| Calendar | spiral calendar | 🗓️ | 0.7612 |
| Leg Injuries | leg | 🦵 | 0.7598 |
| Bandages | adhesive bandage | 🩹 | 0.7590 |
| Fractures, Bone | bone | 🦴 | 0.7570 |
| Lung | lungs | 🫁 | 0.7555 |
| Non-Smokers | no smoking | 🚭 | 0.7539 |
| Mouth | mouth | 👄 | 0.7532 |
| Nematocyst | jellyfish | 🪼 | 0.7522 |
| Students | student | 🧑‍🎓 | 0.7516 |
| Microsurgery | microscope | 🔬 | 0.7510 |
| Thermometers | thermometer | 🌡️ | 0.7486 |
| Microbiota | microbe | 🦠 | 0.7486 |
| Containment of Biohazards | biohazard | ☣️ | 0.7456 |
| Hair Color | person: dark skin tone, curly hair | 🧑🏿‍🦱 | 0.7451 |
| Ear Diseases | ear | 👂 | 0.7445 |
| Foot Diseases | foot | 🦶 | 0.7434 |
| DNA Packaging | dna | 🧬 | 0.7432 |
| Thermometry | thermometer | 🌡️ | 0.7418 |
| Ligation | knot | 🪢 | 0.7414 |
| Health Facilities | hospital | 🏥 | 0.7412 |
| Eye | eye | 👁️ | 0.7408 |
| Fruiting Bodies, Fungal | mushroom | 🍄 | 0.7402 |
| Stomatognathic System | mouth | 👄 | 0.7400 |
| Ocular Physiological Phenomena | eye | 👁️ | 0.7385 |
| Eye Movements | eyes | 👀 | 0.7380 |
| Needles | sewing needle | 🪡 | 0.7372 |
| Musculoskeletal System | bone | 🦴 | 0.7353 |
| Eye Color | eye | 👁️ | 0.7348 |
| Heart | anatomical heart | 🫀 | 0.7329 |
| Bone Diseases | bone | 🦴 | 0.7328 |
| Food and Beverages | canned food | 🥫 | 0.7324 |
| Tomography Scanners, X-Ray Computed | x-ray | 🩻 | 0.7294 |
| Tooth Injuries | tooth | 🦷 | 0.7267 |
| Manuscript | writing hand | ✍️ | 0.7266 |
| Ocular Absorption | eye | 👁️ | 0.7261 |
| Ear | ear | 👂 | 0.7260 |
| Assisted Circulation | beating heart | 💓 | 0.7255 |
| Americas | globe showing Americas | 🌎 | 0.7246 |
| Cheek | face with diagonal mouth | 🫤 | 0.7232 |
| Wool | yarn | 🧶 | 0.7211 |
| Skeleton | bone | 🦴 | 0.7202 |
| Nucleotides | dna | 🧬 | 0.7200 |
| Eye Neoplasms | eye | 👁️ | 0.7193 |
| Microbiological Phenomena | microbe | 🦠 | 0.7181 |
| Nucleosides | dna | 🧬 | 0.7177 |
| Jaw | mouth | 👄 | 0.7175 |
| Central Nervous System | brain | 🧠 | 0.7172 |
| Tooth Diseases | tooth | 🦷 | 0.7169 |
| X-Ray Film | x-ray | 🩻 | 0.7168 |
| Newspaper Article | newspaper | 📰 | 0.7168 |
| Foot Deformities | foot | 🦶 | 0.7163 |
| Hand Injuries | pinching hand | 🤏 | 0.7155 |
| Equipment and Supplies, Hospital | hospital | 🏥 | 0.7155 |
| Mouth Diseases | mouth | 👄 | 0.7151 |
| Neuroimaging | brain | 🧠 | 0.7128 |
| X-Ray Diffraction | x-ray | 🩻 | 0.7115 |
| Transgender Persons | transgender symbol | ⚧️ | 0.7112 |
| Radioactivity | radioactive | ☢️ | 0.7097 |
| Manufacturing and Industrial Facilities | factory | 🏭 | 0.7093 |
| Housing | houses | 🏘️ | 0.7079 |
| Refraction, Ocular | eye | 👁️ | 0.7073 |
| Dental Physiological Phenomena | tooth | 🦷 | 0.7066 |
| Foot Rot | foot | 🦶 | 0.7063 |
| Child, Adopted | child | 🧒 | 0.7057 |
| Lung Diseases | lungs | 🫁 | 0.7053 |
| Host Microbial Interactions | microbe | 🦠 | 0.7052 |
| Chromosomes, Bacterial | dna | 🧬 | 0.7050 |
| Ophthalmologic Surgical Procedures | eye | 👁️ | 0.7045 |
| DNA Replication | dna | 🧬 | 0.7037 |
| Geographic Locations | globe showing Americas | 🌎 | 0.7035 |
| DNA Viruses | dna | 🧬 | 0.7035 |
| Beverages | hot beverage | ☕ | 0.7034 |
| Smokers | cigarette | 🚬 | 0.7028 |
| Health Personnel | health worker | 🧑‍⚕️ | 0.7022 |
| Child, Orphaned | child | 🧒 | 0.7019 |
| Swine Diseases | boar | 🐗 | 0.7015 |
| Acinar Cells | lungs | 🫁 | 0.7014 |
| Transdermal Patch | adhesive bandage | 🩹 | 0.7014 |
| Brain Chemistry | brain | 🧠 | 0.7007 |
| Chart | bar chart | 📊 | 0.7006 |
| Cytological Techniques | microscope | 🔬 | 0.7005 |
